# Genetic characterization of ØVC8 lytic phage for *Vibrio cholerae* O1

**DOI:** 10.1186/s12985-016-0490-x

**Published:** 2016-03-22

**Authors:** Alejandro Solís-Sánchez, Ulises Hernández-Chiñas, Armando Navarro-Ocaña, Javier De la Mora, Juan Xicohtencatl-Cortes, Carlos Eslava-Campos

**Affiliations:** Departamento de Salud Pública, Facultad de Medicina, Universidad Nacional Autónoma de México, Circuito Escolar S/N, Ciudad Universitaria, Delegación Coyoacán, 04510 México, D.F México; Laboratorio de Patogenicidad Bacteriana, Unidad de Hemato-Oncología e Investigación, Hospital Infantil de México Federico Gómez/Facultad de Medicina, UNAM. Dr. Márquez No. 162, Col Doctores, Delegación Cuauhtémoc, 06720 México, D.F México; Departamento de Genética Molecular, Instituto de Fisiología Celular, Universidad Nacional Autónoma de México, Ciudad Universitaria, Delegación Coyoacán, 04510 México, D.F México; Laboratorio de Investigación en Bacteriología Intestinal, Unidad de Hemato-Oncología e Investigación Hospital Infantil de México Federico Gómez, Dr. Márquez No. 162, Col. Doctores, Delegación Cuauhtémoc, 06720 México, D.F México

**Keywords:** *Vibrio cholerae*, Bacteriophage, *Caudovirales*, *Podoviridae*, ØVC8

## Abstract

**Background:**

Epidemics and pandemics of cholera, a diarrheal disease, are attributed to *Vibrio cholerae* serogroups O1 and O139. In recent years, specific lytic phages of *V. cholerae* have been proposed to be important factors in the cyclic occurrence of cholera in endemic areas. However, the role and potential participation of lytic phages during long interepidemic periods of cholera in non-endemic regions have not yet been described. The purpose of this study was to isolate and characterize specific lytic phages of *V. cholerae* O1 strains.

**Methods:**

Sixteen phages were isolated from wastewater samples collected at the Endhó Dam in Hidalgo State, Mexico, concentrated with PEG/NaCl, and purified by density gradient. The lytic activity of the purified phages was tested using different *V. cholerae* O1 and O139 strains. Phage morphology was visualized by transmission electron microscopy (TEM), and phage genome sequencing was performed using the Genome Analyzer IIx System. Genome assembly and bioinformatics analysis were performed using a set of high-throughput programs. Phage structural proteins were analyzed by mass spectrometry.

**Results:**

Sixteen phages with lytic and lysogenic activity were isolated; only phage ØVC8 showed specific lytic activity against *V. cholerae* O1 strains. TEM images of ØVC8 revealed a phage with a short tail and an isometric head. The ØVC8 genome comprises linear double-stranded DNA of 39,422 bp with 50.8 % G + C. Of the 48 annotated ORFs, 16 exhibit homology with sequences of known function and several conserved domains. Bioinformatics analysis showed multiple conserved domains, including an Ig domain, suggesting that ØVC8 might adhere to different mucus substrates such as the human intestinal epithelium. The results suggest that ØVC8 genome utilize the “single-stranded cohesive ends” packaging strategy of the lambda-like group. The two structural proteins sequenced and analyzed are proteins of known function.

**Conclusions:**

ØVC8 is a lytic phage with specific activity against *V. cholerae* O1 strains and is grouped as a member of the VP2-like phage subfamily. The encoding of an Ig domain by ØVC8 makes this phage a good candidate for use in phage therapy and an alternative tool for monitoring *V. cholerae* populations.

**Electronic supplementary material:**

The online version of this article (doi:10.1186/s12985-016-0490-x) contains supplementary material, which is available to authorized users.

## Background

Cholera is a clinical-epidemiologic syndrome caused by ingestion of water contaminated with *Vibrio cholera* serogroups O1 and O139. This disease is considered an important public health problem worldwide, though it mainly affects developing countries and alters the economies of these regions [1]. From 1991 to 2001, the seventh pandemic of cholera affected Latin America, including Mexico. In October 2010, a cholera epidemic in Haiti resulted in over 180,000 cases in three months and spread rapidly to other countries, such as the Dominican Republic and Cuba [2]. *V. cholerae* has the ability to survive in aquatic habitats of different characteristics, including wastewater. During the process of adaptation to conditions of extreme pH, salinity, temperature, and nutrient insufficiency as well as predation by heterotrophic protists and bacteriophages, the expression of different genes is activated. A viable but non-culturable state or biofilm is then induced, which contributes to adaptation by the bacterium for survival in different environmental conditions [3]. Bacteriophages or phages (bacterial viruses) are mobile genetic elements that participate in horizontal gene transfer in bacteria, thereby contributing to their environmental adaptation and evolution. In addition, several bacterial virulence genes are present in phage genomes, and the mobile nature of phages can promote the emergence of new epidemic strains.

One of the main virulence factors of *V. cholerae* is cholera toxin (CT), which is encoded by CTXØ, a lysogenic filamentous phage that has contributed to bacterial evolution through lysogenic conversion and genomic rearrangement [4]. The *ctxAB* genes present in the CTXØ genome of toxigenic *V. cholerae* favor the conversion of nonpathogenic strains into toxigenic strains via CTXØ acquisition. The first vibrio phages were described in 1926 by d’Herelle, and in the 1950s, several distinct types of *V. cholerae* phages were described [5]. The use of bacteriophages as a tool for strain differentiation has contributed significantly to our understanding of cholera epidemiology [6]. In addition, the first phage-typing scheme for *V. cholerae* O1 was employed to study the spread of *V. cholerae* strains of the El Tor biotype [7]. Although, this phage-typing scheme has been used routinely for the classification of *V. cholerae* O1 strains due to its limitations, new phage-typing schemes for O139 strain classification have been developed [8]. Since 2007, more than 200 vibrio phages have been described; however, at present, only 17 genomes of *V. cholerae* phages have been sequenced and annotated in the GenBank database.

In recent years, lytic phages have been proposed as important factors modulating populations of *V. cholerae* serogroups O1 and O139 in the aquatic environment, thus affecting the seasonality and duration of cholera epidemics in endemic areas [9]. In Bangladesh, which is considered an endemic cholera area, the prevalence of several predatory phages (JSF1 to JSF6) of *V. cholerae* has been partially characterized. Fluctuations in and the presence of the most prevalent phage types have been correlated with temporal changes in the cyclical appearance of cholera, acting as factors that modulate the epidemic cycle in the short period as well as outbreak severity [10]. In Mexico, conditions amenable to the survival of *V. cholerae* Non-O1/Non-O139 in aquatic reservoirs have been reported for several years [11]. However, the role of diverse phages in non-endemic cholera areas as elements that participate in the survival and occurrence of the bacterium during long interepidemic periods is not completely understood. In 2010, sporadic cholera cases were identified in Sinaloa State, México, and in 2013, an outbreak of 187 cases of cholera in Hidalgo State, México, was reported by the Secretaria de Salud de México (www.epidemiologia.salud.gob.mx/dgae/boletin/intd_boletin.html; www.sinave.gob.mx/). Although the phages involved in the epidemiology of cholera in Mexico have not yet been characterized, predation of *V. cholerae* O1 by phages can be considered a key factor in understanding the long interepidemic periods of cholera in these regions. The main goal of this study was to isolate and characterize *V. cholerae* phages from wastewater of the Endo Dam in Hidalgo State, México, and to assess their lytic activity against *V. cholerae* O1 strains.

## Methods

### Sampling area

Samples were collected at the Endhó Dam in Hidalgo State, located 80 km north of Mexico City, Mexico. This ~ 1,260-hectare dam has a capacity of approximately 198 million m^3^ and is the main reservoir of wastewater and rainwater from the metropolitan area of Mexico City and Hidalgo State [12].

### Isolation of phages

Four water samples of 200 ml each were collected from different points at the Endhó Dam, transported in glass bottles at room temperature, and processed on the same day. Briefly, 50 ml of each sample was centrifuged at 16,000 × *g* (RC5 rotor, Thermo Scientific, Pittsburgh, PA, USA), and the supernatant was filtered through a 0.22-μm membrane (PVDF; Millipore, Bedford, MA, USA) to eliminate bacteria and/or diverse residues. The phages in the supernatants were isolated using *V. cholerae* O1 and O139 as receptor strains in double-layer plaque assays with soft agar (10 g/l tryptone, 10 g/l NaCl, and 7 g/l agar) as described by Kropinski et al*.* [13] (Table 1). In brief, 1 ml of each sample and 100 μl of the receptor strain in exponential growth phase were mixed, and 4 ml of melted soft agar was added. The mixture was poured onto Petri dishes with nutrient agar (15 g/l agar) and incubated at 37 °C for 18 h.

**Table 1:**
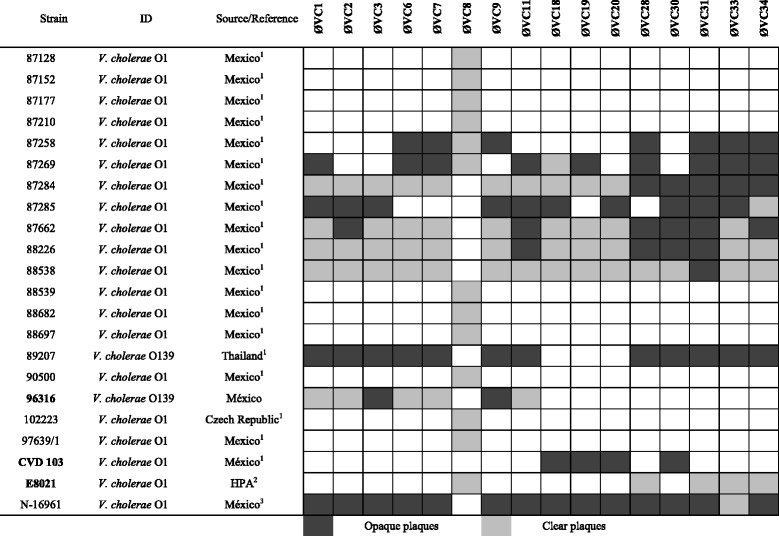
Host range investigated by spot testing

Strains used for initial phage isolation are in bold

1. Strains isolated from different sources were provided by the Laboratory of Bacteriology, Departamento de Salud Pública, Facultad de Medicina, Universidad Nacional Autónoma de México

2. Health Protection Agency, Laboratory of Gastrointestinal Pathogens, London, England

3. Provided by Dr. Shah M. Faruque of the Centre for Food and Water Borne Diseases at the International Centre for Diarrheal Disease Research, Bangladesh (ICDDR)

### Purification of phages

Lytic plaques were selected with a sterile Pasteur pipette and incubated in 50 ml of Luria-Bertani (LB) broth (LB was supplemented with 10 mM CaCl_2_ and inoculated with ~4 × 10^8^ colony-forming units (CFU/ml) of each receptor strain) at 37 °C for 18 h. The cellular debris was eliminated by centrifuging the samples twice at 9,000 × *g*/10 min/4 °C (5415C, Eppendorf, Hamburg, Germany). The supernatants containing the phages were filtered through a 0.22-μm membrane (Millipore PVDF, Bedford, MA, USA). The phages were concentrated adding 5 % polyethylene glycol 8000 (PEG) and 0.25 M NaCl at 4 °C overnight [14] and recovered by centrifugation at 16,000 × *g* (RC5 rotor, Thermo Scientific, Pittsburgh, PA, USA) for 20 min at 4 °C. The pellets were dissolved in 5 ml of SM buffer (50 mM Tris–HCl [pH 7], 100 mM NaCl and 50 mM MgSO_4_), loaded onto a cesium chloride gradient (density, 1.3 to 1.6 g/ml), and centrifuged (41Ti rotor, Beckman, Brea, CA, USA) at 100,000 × *g* for 18 h at 4 °C to obtain relatively pure phages. The recovered phages were dialyzed three times against dialysis buffers “A” (3 M NaCl and 0.1 M Tris–HCl, pH 7.4) and “B” (0.3 M NaCl and 0.1 M Tris–HCl, pH 7.4) using a membrane with a molecular weight cut-off of 12,000 to 14,000 (Spectra/Pore, Spectrum Laboratories, Rancho Dominguez, CA, USA).

### Isolation of V. cholerae

The four water samples previously collected from the Endhó Dam were also used for isolation of *V. cholerae* strains using an enrichment technique [15]. Briefly, 10 ml of each water sample was inoculated into 50 ml of alkaline peptone water and incubated at 37 °C for 6 h. The bacteria-enriched samples were cultured on thiosulfate-citrate-bile salts-sucrose agar (TCBS; Becton-Dickinson, Sparks, MD, USA) at 37 °C for 18 h. Yellow colonies produced by *V. cholerae* on TCBS agar were identified using the GNI-Plus card (bioMérieux, Marcy l’Etoile, Rhône, France) and an automated Vitek system. Strains identified as *V. cholerae* were serotyped with anti-O1 and anti-O139 rabbit sera (Laboratorio de Patógenos Entéricos, UNAM, México).

### Phage host range

The lytic activity of the purified phages was analyzed by a dot plaque assay employing the *V. cholerae* O1, *V. cholerae* O139, *V. cholerae* non-O1, *V. cholerae* non-O139, *V. alginolyticus*, *V. parahaemolyticus*, *V. metschnikovii*, *Aeromonas veronii*, *Escherichia coli* K-12 HB101, and *V. cholerae* isolates identified in this study (Table 1 and Additional file 1: Table S1). To assess phage activity, these strains were cultured on LB agar until log phase and incubated with 10 μl of a phage suspension (10^8^ plaque-forming units [PFU]/ml) at 37 °C for 18 h [16]. The formation of turbid and/or clear plaques over the bacterial lawn was visualized with the naked eye and employed as the criterion for the selection of specific phages.

### Visualization of the ØVC8 phage by transmission electron microscopy (TEM)

Two microliters of a purified phage ØVC8 suspension (1 × 10^8^ PFU/ml) was placed on a Formvar-carbon-coated grid (EMS, Hatfield, PA, USA) for 2 min followed by negative staining with 6 μl of 2 % uranyl acetate for 2 min. The morphology of the purified ØVC8 phage was examined under a JEM 1200 EXII transmission electron microscope (JEOL, Tokyo, Japan).

### Isolation of phage DNA

Phage ØVC8 DNA was extracted as follows: a 500-μl aliquot of phage ØVC8 suspension (1 × 10^8^/ml PFU) was treated with 100 U of *DNase* I (Invitrogen, Carlsbad, CA, USA), 2 μl of proteinase K (20 mg/ml) (Vivantis, Oceanside, CA, USA), and 50 μl of SDS (10 %) at 56 °C for 1 h. The purified DNA was treated with phenol-chloroform (1:1 ratio), precipitated with cold absolute ethanol, and resuspended in DNase-free water.

### Genome sequencing and assembly of phage ØVC8

Total phage ØVC8 DNA was sequenced using Genome Analyzer IIx System (Illumina, San Diego, CA, USA) at the Massive Sequencing University Unit (UUSM, by its initials in Spanish) at the Institute of Biotechnology of UNAM (Cuernavaca, Morelos, Mexico). *De novo* assembly of ØVC8 was performed at Winter Genomics Company (www.wintergenomics.com) using the Velvet, Abyss, and SOAP programs. A consensus sequence from the results obtained with three programs was generated using the Minimus program [17].

### Bioinformatics analysis of the ØVC8 genome

The consensus sequence of the ØVC8 genome was analyzed as described by Henn et al*.* [18]. To identify potential coding sequences, the programs BLAST-X and PFAM/TIGR were used to compare the ØVC8 genome sequence against the sequences of proteins reported in the databases. Open reading frames (ORFs) identified using the Glimmer3, Meta GeneAnnotator, GeneMarkS, ZCURVE_V, and EasyGene gene prediction programs were grouped as a single locus. tRNAs and rRNAs were predicted using the tRNAscan-SE, Rfam, and ARNmmer programs. The pI/MW program was used to calculate the molecular weights of the identified proteins [19]. The transcriptional promoters and terminators were predicted using the PromoterHunter and WebGeSTer programs, respectively [20, 21].

### Phylogenetic analysis of ØVC8

A phylogenetic analysis of ØVC8 was performed using the translated DNA sequence of ORF3 (terminase large subunit), which was aligned with 53 homologous sequences from phages of the *Podovirus* family using the ClustalW2 program [22]. Additionally, the phylogenetic tree was constructed with the Mega ver. 6.0 program using the neighbor-joining method, which employs a gamma distribution (gamma = 2) and 1,000 bootstrap replicates with Poisson distance correction [23].

### Accession number of the nucleotide sequence

The genome sequence and the genetic annotation of genome ØVC8 were deposited in the GenBank database under accession number JF712866.

### Structural proteins of phage ØVC8

Potential proteins in the phage ØVC8 capsid were identified according to the procedure described by Boulanger et al. [24]. The phage was precipitated with PEG/NaCl (as described above), mixed with Laemmli solution (65.8 mM Tris–HCl, [pH 6.8], 2.1 % SDS, 26.3 % [w/v] glycerol, 0.01 % bromophenol blue and 100 mM β-mercaptoethanol) and heated to 100 °C for 5 min. Proteins were separated by 10 % SDS-PAGE (polyacrylamide-sodium dodecyl sulfate gel electrophoresis) and visualized using Coomassie blue. The identified proteins were processed using a QTRAP 3200 mass spectrometer (Applied Biosystems/MDS Sciex, ON, Canada) at the Biochemistry Department of the Faculty of Medicine-UNAM.

## Results

### Isolation of bacteria and phages

Thirteen isolates identified in wastewater samples from the Endhó Dam were characterized as *V. cholerae* non-O1/O139 (6 isolates), *V. alginolyticus* (4 isolates), and *A. veronii* (3 isolates) (Additional file 1: Table S1). In addition, 16 phages were isolated from these wastewater samples.

### Lytic activity and host specificity of the identified phages

The sixteen phages identified were tested using 53 gram-negative bacteria; although 58.49 % (31/53) of these bacteria were not infected by any phage (Table 1 and Additional file 1: Table S1). On the other hand, all the phages showed lytic and lysogenic activity when tested against 22 *V. cholerae* O1/O139 strains. In this assay, 75 % (12/16) of the phages produced clear and/or opaque plaques in both serotypes (*V. cholerae* O1 and O139), though four of the phages did not infect *V. cholerae* O139 (Table 1). Interestingly, only the phage designated as ØVC8 showed lytic activity against thirteen strains of *V. cholerae* O1, producing clear plaques ~1 mm in diameter without halos.

### Morphology of the ØVC8 phage

**Fig. 1:**
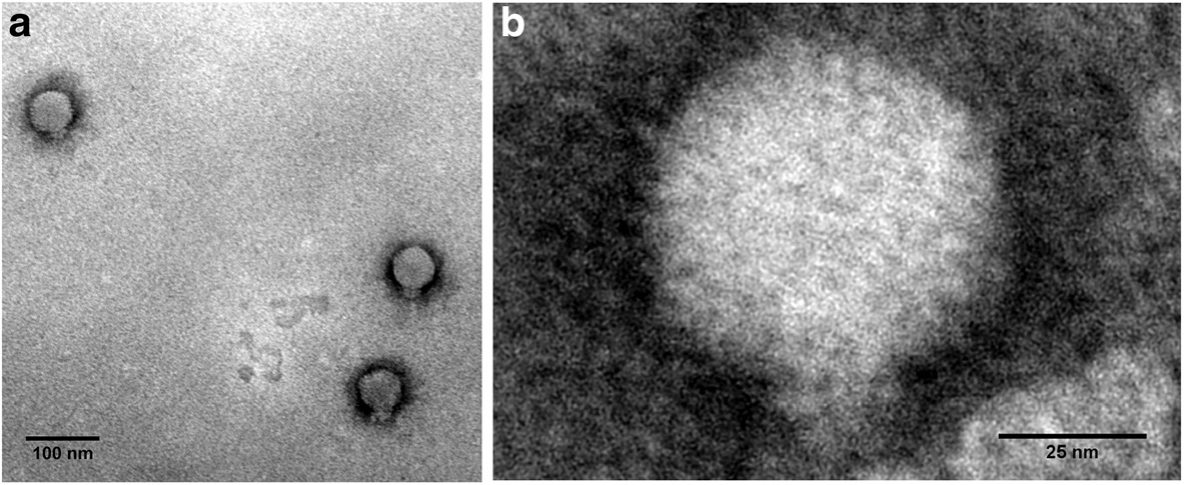
Electron microscopy of the ØVC8 phage isolated from water samples obtained at the Endhó Dam. Micrograph of a negatively stained sample showing three ØVC8 phages (**a**). Micrograph showing a zoomed-in of a phage (**b**). The microphotograph shows the hexagonal capsid and the short tail of ~16 nm in detail; both are characteristics of the *Podoviridae* family. The phage was negatively stained with uranyl acetate (2 %). Magnification: 50,000 ×

The ØVC8 phage was stained with 2 % uranyl acetate and analyzed by TEM. Morphological analysis showed an isometric icosahedral capsid approximately 62 nm in diameter and a tail 16 nm in length (Fig. 1a and b). These characteristics are similar to the phages described in the *Podoviridae* family of the order *Caudovirales*.

### Genome sequencing of ØVC8

The genomic sequence of ØVC8 was assembled into a single contig with a median coverage of 14,324×, composed of a double-stranded DNA molecule 39,422 bp in length with 50.8 % G + C content. Bioinformatics analysis of the ØVC8 genome sequence revealed 48 putative ORFs; of these, 30 % (14/48) can be assigned functions according to their homology to known sequences of other phages, 2.08 % (1/48) do not show similarity with any previously reported sequences, and 70.83 % (34/48) correspond to hypothetical proteins described in other phages (Table 2). The 48 ORFs are distributed on both DNA strands: one strand contains 22 ORFs related to phage packaging functions and to mainly structural proteins, and the other 26 ORFs on the complementary strand are associated with metabolic, replication and unknown functions. tRNAs and rRNAs were not identified.

**Table 2:** Putative open reading frames in the ØVC8 genome and their assigned functions

			Gene product						
ORF	Nucleotide position		Size	^a^ Molecular mass	Presumed function	Related phage or organism	GenBank accession no	BLAST X	
	Start	End	(aa)	(kDa)				% Identity	e-value
1	1	222	73	13.6	Hypothetical protein	Vibrio phage VP2	YP_052974	95	1 e-32
2	128	739	203	22.9	Terminase small subunit	Vibrio phage VP5	YP_053007	94	4 e-131
3	726	2432	568	65.3	Terminase large subunit	Pelagibacter phage HTVC010P	YP_007517700	32	4 e-41
4	2443	4086	547	61.7	Head-to-tail connecting protein	Pelagibacter phage HTVC010P	YP_007517703	32	4 e-62
5	4086	4313	75	8	Hypothetical protein	Vibrio phage VP2	YP_052976	92	7 e-26
6	4326	4583	85	9.9	Hypothetical protein	Vibrio phage VP2	YP_052977	95	2 e-18
7	4552	5439	295	31.8	Hypothetical protein	Pelagibacter phage HTVC010P	YP_007517705	23	6 e-14
8	5607	6575	322	36.1	Structural protein	Pelagibacter phage HTVC010P	YP_007517707	24	1 e-21
9	6642	6911	89	9.2	Hypothetical protein	Vibrio phage VP2	YP_052980	100	7 e-35
10	6926	7774	282	31.1	Hypothetical protein	Vibrio phage VP5	YP_053013	91	1 e-46
11	7771	8136	121	13.4	Hypothetical protein	Vibrio phage VP2	YP_052982	96	1 e-59
12	8138	8611	157	18.2	Hypothetical protein	Vibrio phage KVP40	NP_899538	31	2 e-20
13	8604	8906	100	10.5	Hypothetical protein	Vibrio phage VP2	YP_052983	97	1 e-51
14	8930	11155	741	74.1	Tail protein	Vibrio phage VP5	YP_024980	90	0
15	11164	12948	594	65.5	Structural protein	Vibrio phage VP5	YP_024422	98	0
16	12951	13349	132	13.9	Hypothetical protein	Vibrio phage VP2	YP_052984	97	1 e-41
17	13346	15430	694	76.8	Structural protein	Vibrio phage VP2	YP_052985	95	0
18	15430	16602	390	41.5	Hypothetical protein	Vibrio phage VP5	YP_053019	95	2 e-151
19	16604	18952	782	87.2	Structural protein	Escherichia phage phiV10	YP_512274	27	6 e-68
20	18956	19984	342	36.6	Tail fiber	Vibrio phage VP5	YP_053020	96	2 e-162
21	20009	21322	437	44.9	Outer capsid protein	Vibrio phage VP2	YP_024425	31	1 e -35
22	21350	21646	98	11.2	Hypothetical protein	Pseudomonas phage PPpW-3	YP_008873205	45	9 e-16
23	21723	22160	145	11.2	Hypothetical protein	Vibrio phage VP2	YP_053022	96	2 e-79
24	22150	22308	52	11.2	Hypothetical protein	Vibrio phage VP2	YP_052988	81	1 e-19
25	22305	22817	170	19.2	Metal dependent phosphohydrolase	Vibrio phage VP5	YP_024983	94	7 e-116
26	22819	23079	86	9.8	Hypothetical protein	Vibrio phage VP5	YP_053024	96	2 e-28
27	23081	24112	343	38.1	Adenylosuccinate synthetase	*Solibacter usitatus*	YP_827486	33	1 e-31
28	24154	26442	762	87	Integrase	Vibrio phage VP2	YP_024428	99	0
29	26432	28333	633	71.2	DNA Polymerase I	α-proteobacteria phage ØJL001	YP_223952	26	9 e-40
30	28330	28680	116	13.5	Hypothetical protein	Enterococcus phage EFDG1	AJP61480	53	8 e-14
31	28677	29192	171	19.2	ssDNA binding protein	Vibrio phage VP2	YP_024430	93	8 e-95
32	29253	30068	271	30.3	Hypothetical protein	Pseudomonas phage F8	YP_001294468	32	1 e-23
33	30119	30586	155	17.8	Hypothetical protein	Vibrio phage VP2	YP_052991	98	5 e-108
34	30649	32118	489	55.4	Superfamily II DNA/RNA helicases	Thermoanaerobacterium phage THSA-485A	YP_006546319	36	2 e-77
35	32188	32538	116	12.6	Hypothetical protein	Vibrio phage VP5	YP_053027	84	1 e-26
36	32538	33230	230	24.5	Hypothetical protein	Vibrio phage CJY	AIZ01434	97	3 e-111
37	33381	33845	154	17.3	Hypothetical protein	Vibrio phage VP2	YP_052993	84	6 e-93
38	33842	34252	136	15.7	Hypothetical protein	Vibrio phage VP2	YP_052994	78	2 e-64
39	34254	34613	119	13.7	Hypothetical protein	Vibrio phage VP5	YP_053031	67	4 e-44
40	34621	35421	266	30.2	Hypothetical protein	Vibrio phage VP2	YP_052996	98	2 e-101
41	35490	35975	161	18.1	Hypothetical protein	Vibrio phage VP2	YP_052977	96	5 e-85
42	35985	36437	150	16	Hypothetical protein	Vibrio phage VP5	YP_053036	84	2 e-61
43	36543	36836	97	11	Hypothetical protein	Vibrio phage VP2	YP_053001	95	1 e-46
44	36848	37138	96	10.7	Hypothetical protein	Vibrio phage VP2	YP_053002	79	5 e-36
45	37245	37490	81	9.2	Hypothetical protein	Vibrio phage VP2	YP_053003	74	1 e-26
46	37495	38094	199	22.1	Hypothetical protein	Vibrio phage VP5	YP_053042	91	5 e-93
47	38093	38788	231	26.9	Hypothetical protein	Vibrio phage VP2	YP_053005	93	1 e-50
48	38669	39028	121	13.6	Hypothetical protein	Unknown	---	---	---

^a^Predicted using the Compute pI/Mw tool, Swiss Institute of Bioinformatics

### Functional organization of the ØVC8 genome

**Fig. 2:**
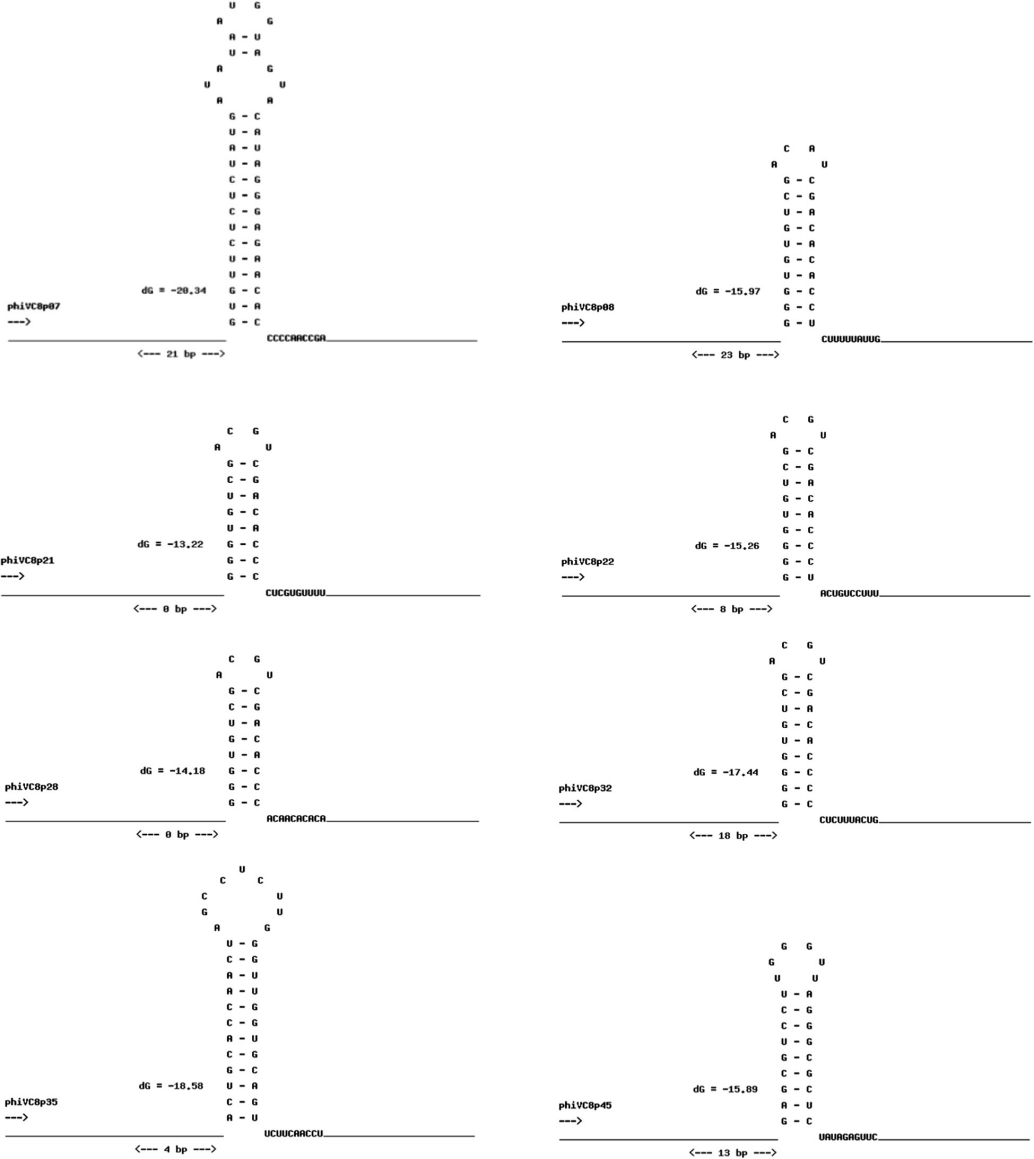
Putative sequences and predicted secondary structure of Rho-independent terminators found in the ØVC8 genome using the WebGeSTer program. The ORF-associated terminator is indicated to the left of each secondary structure; the distance separating the terminator from the stop codon and the free energy of the secondary structure (dG) are also provided

**Fig. 3:**
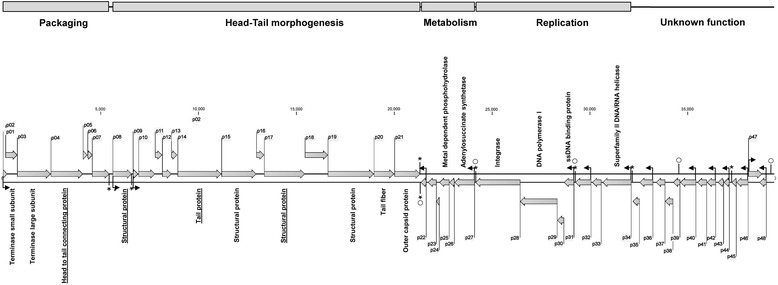
ØVC8 genome organization. The start site was arbitrarily assigned to ORF 1. Arrows represent predicted genes and transcription direction. Promoter positions and directions are indicated by thin arrows; Rho-independent terminators, by asterisks; repeat sequences, by open circles; and underlined ORFs were also determined by mass spectrometry. Rectangles represent the proposed functional modules for the ØVC8 genome

Hypothetical regulatory sequences were found in the intergenic regions of the ØVC8 genome: 15 correspond to promoter sequences (Table 3), and eight are associated with Rho-independent terminators (Fig. 2). Considering the specific regulatory sequence positions and ORF functions, the ØVC8 genome is organized into functional modules of packaging, head-tail morphogenesis, metabolism, and replication (Fig. 3). Additionally, two possible modules (ORFs 48 to 45 and 44 to 35) described as hypothetical proteins without assigned functions are located upstream of the replication module. Furthermore, five repeat sequences are present in the genome: four of these are inverted sequences of 20 bp, three are located in intergenic regions of both replication and metabolism modules, and the last is located in ORF39 (hypothetical protein). Additionally, a tandem direct repeats sequence of 54 bp was also found in the non-coding region 222 bp upstream of ORF48.

**Table 3:** Predicted promoter sequences found in intergenic regions in the ØVC8 genome using the PromoterHunter program

Promoter	ORF	Position	5'	- 35	- 10	3'
1	1	39373…39422	ATGTAACGGT-**GGTTGACA**-CAGAGCCAGAGGTGTG-**CTATAAT**-GGAGGTAAG			
2	8	5551…5601	AGAGTCCGGA-**AACGGGTA**-GCTCAAATCGATATTA-**ACAATCT**-TTTAGGAAAA			
3	9	6593…6641	GATTAGGG-**TGTGTCGACA**-TCGACACACCTCTTT-**TTATTGG**-AGAATACAT			
4	22	21652…21701	AACCCTTGA-**TTTTTTACC**-GCACCGTGAAGGGTG-**CAAAGAG**-GCAGGAGAAA			
5	27	24113…24153	AAGAATAGGG-**TGTCGACG**-TCGACACCCACAAC-**ACACAAGAG**-GAAACACA			
6	31	29193…29235	GGGGTGTCGA-**CGTCGACA**-CCCCCTCTTTACTGG-**AGAGACT**-TAA			
7	32	30075…30118	C-**AAGTAACG**-AAGCAACTAGTAACGAAA-**TAACTGT**-CAATGGAGAC			
8	34	32127…32178	CCAACTAGCC-**TCTTGGGT**-TGGTGCAGTTCTTCAAC-**CTAACCC**-AATGCAAGCA			
9	36	33231…33280	TGTCGCCCAC-**CCTTTAGC**-TATCCAACCAAACCA-ATCGTAG-GAGCACGACG			
10	40	35422…35472	CTGCAATAGA-**GCTTGACG**-CGCGCCCATATGTATGTT-**ATAATAG**-GTGTGTCG			
11	42	36449…36500	TTGTCGCAAT-**CTTTTATT**-TGGGGGAGGGCGTGTCC-**CTCCTGT**-CTCAACTAGT			
12	44	37139…37158	CAATGGTGTC-**GACGGTCG**-ACACACCCCGAACAC-**GTAAGGA**-GTCCGAA			
13	46	38150…38200	TTGCAAGGGT-**ACTTGACA**-GGCATCCGAAAGTGTG-**TTAAAAT**-AAGAACACAA			
14	47	38040…38092	CTTGCTTGCT-**GACCACCA**-CTCTGGTGTTTAGTTCGT-**AGCTGCT**-TGTGCTGCTC			
15	48	39031…38080	TCGGTCACAA-**AATGTTCG**-AAATGTGACAAAACT-**CACATCT**-TTGACACACG			

Sequences in bold correspond to −10 and −35 regions

### Packaging module

**Fig. 4:**
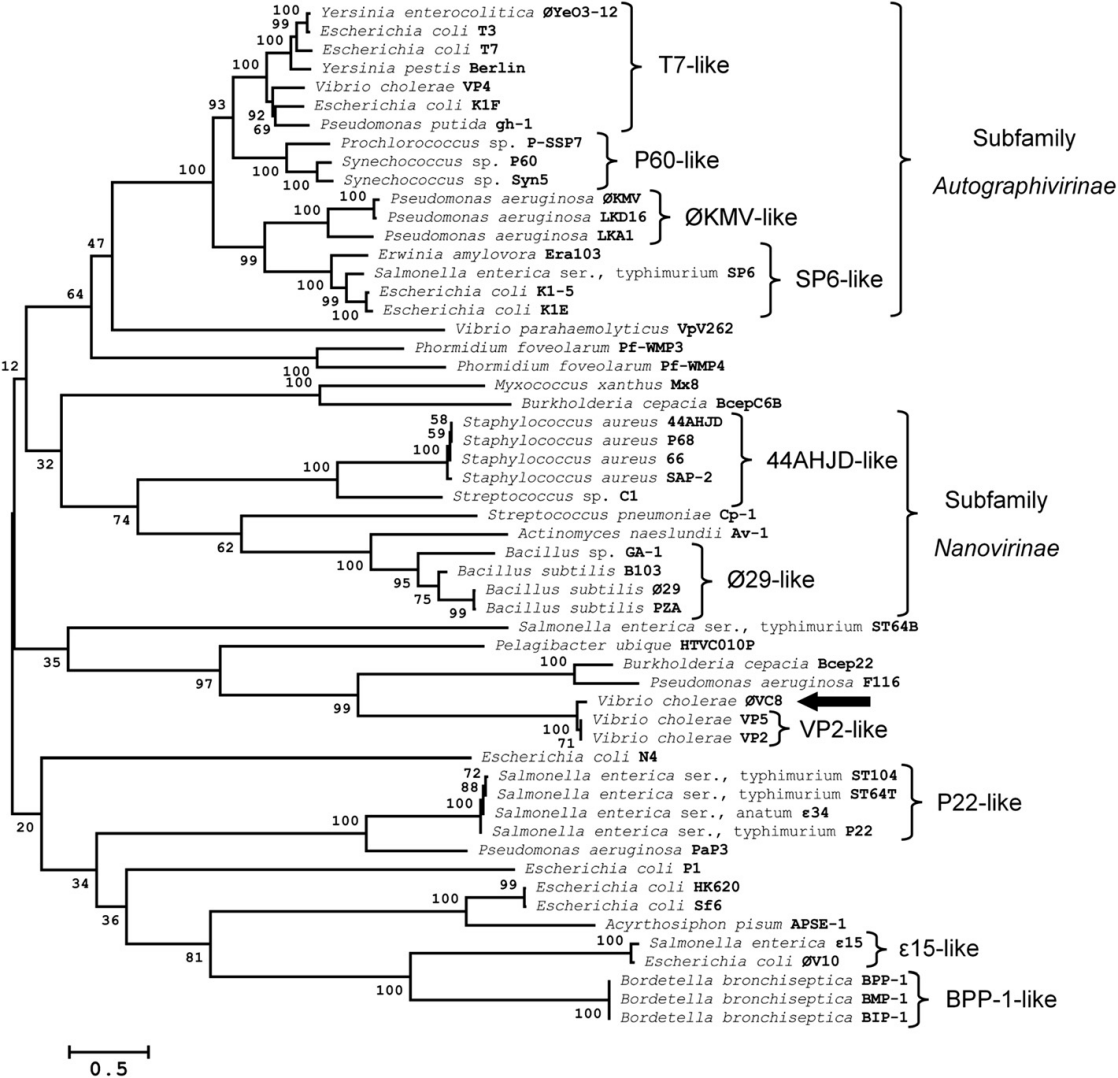
Neighbor-joining tree for comparing the amino acid analysis of the terminase large subunit (ORF3) of ØVC8 and derived sequences in GenBank from 54 other phages of the *Podoviridae* family. The keys represent the major groups, and the respective hosts followed by the phage name are included at the end of each branch. Black arrow indicates the group of ØVC8 phage. The numbers in the internal nodes are bootstrap values (0-100 %) obtained from 1,000 bootstrap replicates

Seven ORFs were identified in this module, four of which are annotated as hypothetical proteins with unknown functions. Functions for ORFs 2, 3, and 4 can be ascribed to the small terminase subunit, large terminase subunit, and head-to-tail connecting protein, respectively (Fig. 3). The functional characteristics of the three ORFs and closely related homologs are described in Table 2. Characterization of the large terminase subunit as a protein widely conserved in the *Podoviridae* family is important for determining the close phylogenetic relationship of ØVC8 with *V. cholerae* phages VP2 and VP5 (Fig. 4).

### Head-tail morphogenesis module

Fourteen ORFs are located downstream of the packaging module, seven (ORFs 9 to 13, 16, and 18) of which are described as hypothetical proteins. In addition, five ORFs (ORFs 14, 15, 19, 20, and 21) were identified using BLAST-X, and two (ORFs 8 and 17) were identified by mass spectrometry. The hypothetical proteins of these ORFs show homology to structural proteins of the VP2 phage (Table 2). ORF14 codes for a 74.1-kDa protein similar to the tail protein of VP5 (identity = 90 %) and contains conserved traits of specific domains of the immunoglobulin (Ig) superfamily. ORF21 shows 96 % identity with the capsid protein of VP2*.* Pfam analysis of the ORF21 sequence reveals a BNR/Asp-box repeat conserved domain of the bacterial neuraminidase or sialidase family.

### Replication module

Seven ORFs (34 to 28) located at the 3′ end of the complementary strand encode proteins involved in ØVC8 phage replication. ORFs 34, 31, 29 and 28 are annotated as a helicase, single-strand DNA-binding protein (SSBP), DNA polymerase I, and integrase functions, respectively (Fig. 3 and Table 2). ORF34 exhibits an amino-terminal conserved region with an SNF-2 domain that corresponded to the helicase-like ATP-dependent family. ORF31, which is located downstream, shows 93 % identity with SSBPs of phage VP2. ORF29 codes for DNA polymerase I, which has also been described in T3 of *Myoviridae*, T5 of *Siphoviridae*, and T7 of *Podoviridae*. ORF28 encodes an integrase of VP2 and VP5 phages, with 99 and 56 % identity, respectively. A bifunctional-N-terminal primase/polymerase (N-Ter prim/pol) and a primase C-terminal-2 domain (PriCT-2) in ORF28 were also identified. ORFs 33, 32, and 30 are hypothetical proteins.

### Metabolism module

The ØVC8 metabolism module is composed of six ORFs (22 to 27 ORFs). The sequences of ORFs 25 and 27 are enzymes involved in the metabolic pathways of amino acid synthesis. ORF25 encodes an HD-3 conserved domain with an H-21x-HD motif that corresponds to a metal-dependent phosphohydrolase from the HD superfamily, and ORF27 encodes an adenosyl succinate synthetase. ORFs 22, 23, 24, and 26 are hypothetical proteins (Fig. 3).

### Comparative genomics of ØVC8 with VP2 and VP5

The BLASTX alignment results for the ØVC8 genome revealed 86 and 85 % identity compared with the VP2 and VP5 genomes, respectively [25]. Differences at the DNA sequence level include six regions of the ØVC8 genome compared with the VP2 genome and four regions compared with the VP5 genome. Consistently, the putative proteins of six ORFs localized in these regions of the ØVC8 genome display sequence similarities with different percentages of identity and positions compared to VP2 and VP5 phage proteins (Additional file 2: Figure S1). Interestingly, the putative protein of ORF30 is not present in the VP2 and VP5 genomes.

### Phylogenetic analysis of the terminase large subunit

**Fig. 5:**
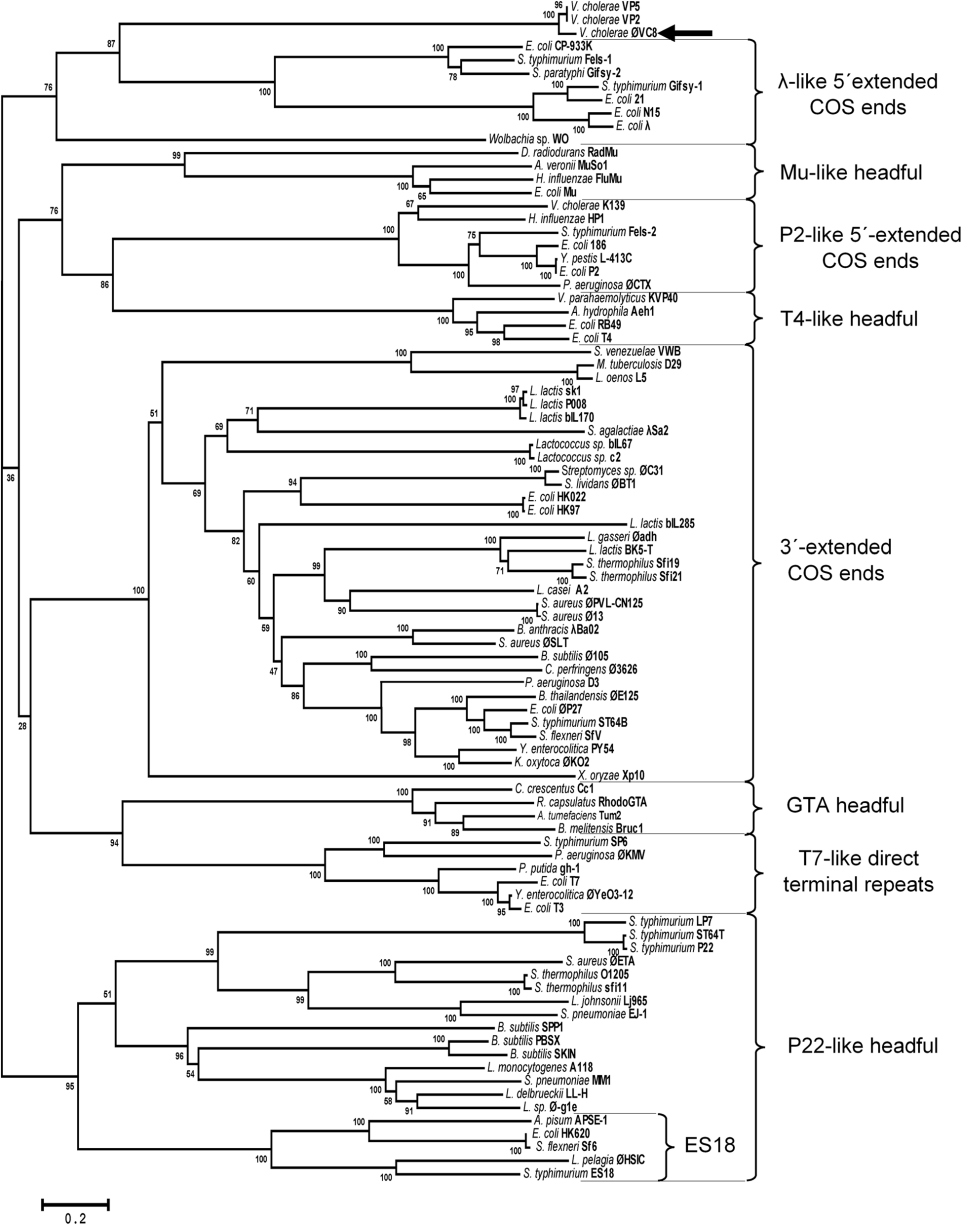
Neighbor-joining tree for comparing the amino acid sequence of the large terminase subunit of ØVC8 (ORF3) and 88 phages of the order *Caudovirales*. Major related groups of terminases are marked with brackets and dotted lines. Black arrow indicates the group of ØVC8 phage. The DNA packaging strategy and phage type for each group are indicated to the right of each bracket

To investigate the possible role of the terminase subunit in the ØVC8 genome packaging process, bioinformatics analysis comparing the amino acid sequence of ORF3 with sequences of 88 phages of the order *Caudovirales* was performed. The phylogenetic tree grouped ØVC8 ORF3 into the same cluster with the terminases of VP2, VP5, CP-933 K, Fels-1, Gifsy-1, Gifsy-2, and Wo phages from the lambda-like group (Fig. 5).

### Structural proteins

**Fig. 6:**
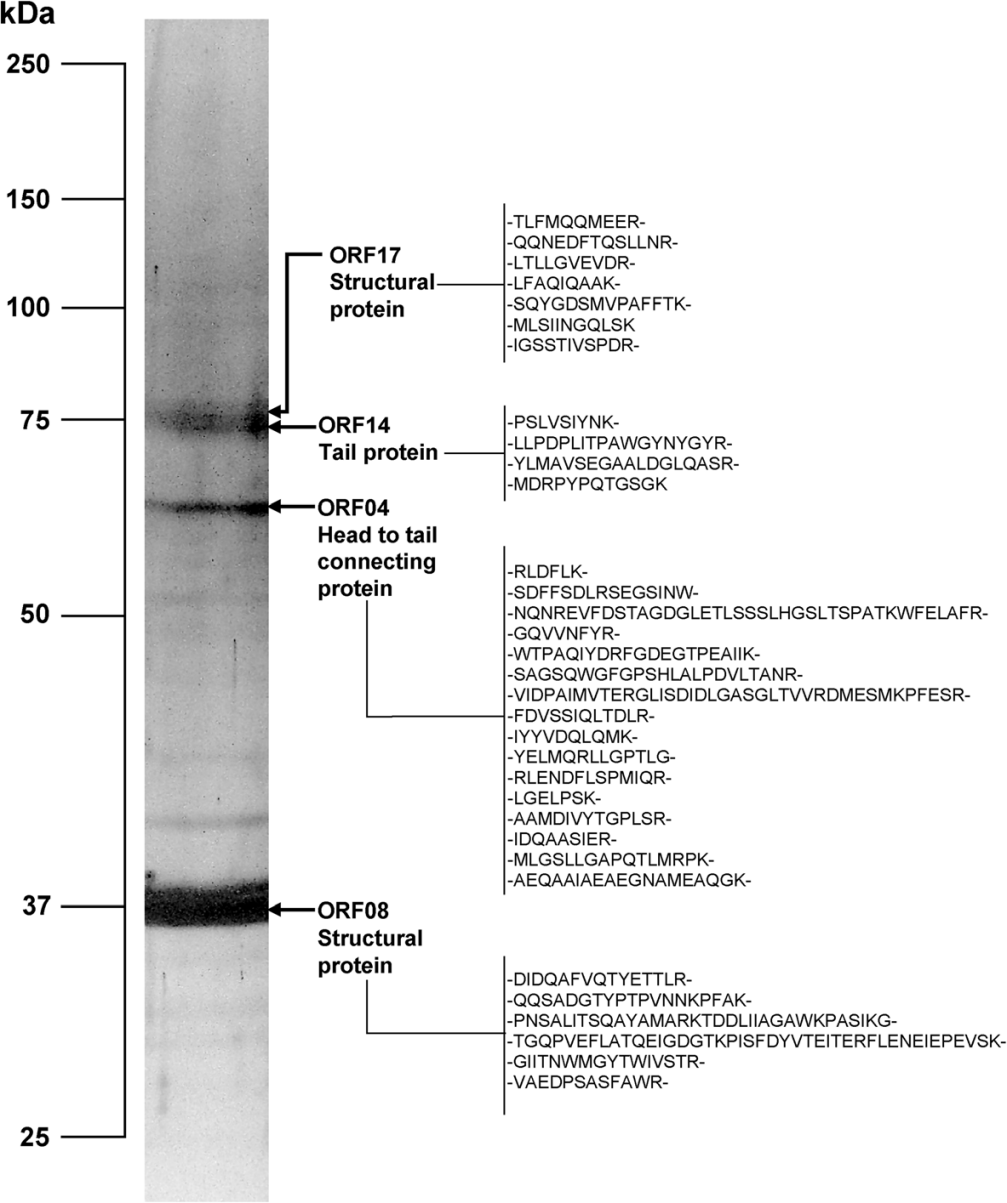
SDS-PAGE (12 %) analysis of ØVC8 proteins. Peptide N-terminal sequences of four structural proteins of ØVC8 were obtained by mass spectrometry. BLAST-P analysis showed that one protein (61.7 kDa) matches with the head-to-tail connecting protein and another (74.1 kDa) with the tail protein; both have been reported in VP2 and VP5 vibrio phages. The other two proteins (36.1 and 76.8 kDa) are annotated as hypothetical proteins

Structural proteins of ØVC8 phage were purified, analyzed, and compared with 48 ORFs of the ØVC8 phage genome (Table 2). Four main proteins (36.1, 61.7, 74.1, and 76.8 kDa) were identified by SDS-PAGE analysis and sequenced by mass spectrometry (Fig. 6). BLAST-P analysis of the amino-terminal sequences of the 61.7 and 74.1 kDa proteins showed matches to structural proteins of phages VP2 and VP5. In addition, 61.7 kDa protein peptides are homologous to specific peptides of head-to-tail (ORF4), and 74.1 kDa protein peptides are homologous to specific peptides of the tail protein (ORF14). In contrast, matches with hypothetical proteins of unknown functions were found for the 36.1- and 76.8-kDa proteins, corresponding to ORFs 8 and 17; these proteins have been described in the VP2 and VP5 phages.

## Discussion

Phages as biological entities are abundant and widely distributed in the world and have great relevance in the control of bacterial communities. Fluctuations in phage populations during the seasonal behavior of cholera and the surveillance of *V. cholerae* in the aquatic environment are important factors that have been associated with cholera outbreaks [9]. In endemic cholera regions, *V. cholerae* phages have been detected in high frequency in different aquatic habitats, and these phages have been employed as strain markers for phage typing of *V. cholerae* O1 and O139 [8].

Thirteen bacterial isolates in wastewater samples collected from the Endhó Dam in Hidalgo State were identified as *V. cholerae* non-O1/O139, *V. alginolyticus* and *A. veronii*. Although toxigenic isolates of *V. cholerae* O1/O139 were not identified, the presence of non-O1/O139 *V. cholerae* strains is suggestive of the ability of these bacteria to survive for prolonged periods in sewage-polluted waters.

In endemic cholera areas, the presence of non-O1/O139 strains in the environment has been related to these bacteria serving as possible phage reservoirs with lytic activity against *V. cholerae* O1/O139 [9]. An abundant number of phages in wastewater treatment systems have been described, though little information regarding their population dynamics and their interaction with the microbial community has been published [26]. In the present work, only one phage, named ØVC8, showed lytic activity again *V. cholerae* O1 strains. *V. cholerae* predation by lytic phages has been proposed to be an important factor involved in the cyclical occurrence and severity of cholera outbreaks in endemic areas [10]. Thus, the presence of ØVC8, a lytic phage of *V. cholerae* O1 strains, could be involved in the epidemiology of cholera in Mexico, possibly regulating the presence of *V. cholerae* O1 strains for long periods; however, further studies are required to confirm this possibility.

The morphological characteristics of ØVC8 phage visualized by TEM showed a structure similar to *V. cholerae* phages JSF3 and JSF6 of the *Podoviridae* family, two phages that have been associated with the cyclic appearance of cholera in Bangladesh (Fig. 1). Considering the morphological classification of JSF3 and JSF6 phages, ØVC8 could be included in group III of the *V. cholerae* phage C1 morphotype, which includes OXN-100P, 4996, I, and III [5–27].

Sequencing of the ØVC8 genome revealed 48 putative ORFs distributed on both DNA strands and organized into packing, head-tail morphogenesis, metabolism, replication, and unknown function modules. ORFs 2 and 3 encode for proteins of the terminase family, which are implicated in translocation of the viral capsid DNA during the final stage of phage assembly. Terminases are the most conserved proteins among caudoviruses, and they have been identified in all podoviruses [28]. Considering the presence of terminases as a potential marker of podoviruses, ORF3 of phage ØVC8 was analyzed by bioinformatics procedures. The results obtained allowed the identified terminases to be grouped in the same cluster with the terminases of phages VP2 and VP5 (Fig. 4). These data support that ØVC8 has a genomic organization that is similar to that of VP2 and VP5; therefore, ØVC8 could be included in the VP2-like subfamily proposed by Lavigne et al*.* [29].

During ØVC8 phage replication, a terminase protein is required for DNA packaging and for chromosomal end formation [30]. ORF3 encodes a large terminase subunit that could participate in this process. Indeed, comparative analysis of the terminase protein coded by ORF3 suggests participation in the packing process via the “single-stranded cohesive ends” strategy, as has been described for lambda-like phages. Phages with these characteristics have a complementary sequence and generate protruding single strands called COS sites; these sites are highly conserved in the genome and are present in a region 1,000 bp upstream of the gene that encodes the small terminase subunit [28]. The above findings led us to propose that the COS site of ØVC8 is located in a tandem sequence 299 bp upstream of the terminase small subunit (Fig. 3). The interaction of head-to-tail connecting proteins with one of the procapsid vertices of the mature phage promotes formation of an axial pore for DNA translocation in both directions [31]. The presence of the head-to-tail connecting protein (ORF4) of ØVC8 suggests that this protein participates in this process during DNA packaging by translocating the chromosome into the procapsid and ejecting it during the infective stage of the phage. These results indicate that ORF4 is the portal protein of phage ØVC8.

In some podovirus phages such as P22 from *Salmonella* sp., tail proteins have been described as molecules with the ability to recognize specific receptors during the initial stages of host infection [32]. In the head-tail morphogenesis module of ØVC8, ORF14 encodes a tail protein that could be involved in the recognition and infection of *V. cholerae* O1 strains. However, an Ig domain similar to that described for phage T4, which has been associated with functions of immune response and adhesion to eukaryotic cells, was also identified in ORF14 [33]. This domain has been grouped into the classic Ig domain (I-Set), which is widely distributed among bacteria, as well as the fibronectin type 3 (FN3) and bacterial Ig-like domain (Big2) families. Bioinformatics analysis of ORF14 revealed that its Ig domain corresponds to the I-Set family [34]. Recent studies have demonstrated that Ig-like domains are important in phage interaction with metazoan mucosal surfaces via specific adherence that might provide immunity independent of the host immune response [35]. Thus, the Ig domain of ORF14 may be an important element in ØVC8 phage interaction with the human intestinal mucosa, which is associated with the lytic activity of the phage in preventing *V. cholerae* O1 colonization.

Although ORF20 codes for a tail fiber protein, the presence of fibers in the ØVC8 phage was not observed by TEM. The tail fiber protein described for phage T7 consists of elongated homo-trimers that are responsible for the reversible initial recognition of a cell [36]. These structures are commonly composed of six fibers that are attached to the phage capsid, which hinders TEM analysis, and these fibers can be only visualized when host interaction occurs [37]. In our study, ØVC8 phage fibers were not observed, suggesting the possibility of a situation similar to that described for T7. In contrast, ORF21 of ØVC8 was annotated as a capsid protein, which encodes a BNR/Asp-box domain that has been described in the neuraminidase or sialidase family from bacteria and phages [38]. Proteins with sialidase activity are important for the degradation of bacterial polysaccharides; expression of these enzymes is an attractive feature for phage therapy [39]. Indeed, the presence of the BNR/Asp-box domain in ORF21 enables ØVC8 to be considered as a possible strategy for the treatment of cholera.

DNA/RNA helicases are widely distributed proteins that are required for the ATP-dependent unwinding of double-stranded DNA, an essential step in replication, expression, recombination, and DNA repair. In the replication module of the ØVC8 genome, ORF34 encodes a DNA/RNA helicase of the SNF-2 family with a conserved domain in its amino-terminal region that is involved in chromatin structure remodeling [40]. ORF31 codes for SSB, a protein that participates in replication, recombination and DNA repair processes [41]. ORF29 encodes for a DNA polymerase I described in mitochondrial polymerase-g, prokaryotic DNA polymerase I, and diverse polymerases (T3, T5, and T7 phages) of the Pol A family [42]. Therefore, the helicase of ORF34 identified in this study may participate in transcription and replication processes of the ØVC8 phage genome.

ORF28 exhibits sequence homology with integrases of *V. cholerae* phages VP2 and VP5*.* Integrases achieve viral genome integration into the host genome via site-specific recombination of DNA sequences of 30 to 40 bp, with the first located on the phage chromosome (attP) and the second on the bacterial chromosome (attB). These enzymes are classified into two major families based on their amino acid sequence homology and catalytic residues, either tyrosine or serine. However, bioinformatics analysis shows no tyrosine or serine residues in the sequence of ØVC8 integrase or the corresponding sequences of VP2 and VP5 phages. Suggesting that the sequence does not correspond to an integrase or is a non-functional protein. Conserved bifunctional-N-terminal primase/polymerase domains (N-Ter prim/pol) and other primase C-terminal-2 domains (PriCT-2) were identified in the ORF28 sequence of the ØVC8 genome. N-Ter prim/pol is a multifunctional enzymatic domain with ATPase, primase, DNA polymerase, and helicase activity [43]. In contrast, the PriCT-2 domain belongs to the archaea-eukaryotic primase superfamily from the primase-polymerase clade (prim/pol-like) [44]. N-Ter prim/pol and PriCT-2 are essential domains of multifunctional replication proteins of the phage replication machinery. Accordingly, we speculate that ORF28 has bifunctional DNA primase/polymerase activity involved in ØVC8 phage replication.

In the metabolism module of the ØVC8 genome, ORFs 25 and 27 encode for enzymes involved in metabolic pathways of amino acid synthesis; these proteins are auxiliary metabolic molecules that may provide additional support in host metabolism steps, allowing successful phage infection [45]. HD-3, a conserved domain of ORF25, corresponds to proteins with a distinct combination of metal-chelating residues, nucleases and phosphodiesterase activities [46]. These data suggest that ORF25 could participate in ØVC8 phage signaling and nucleotide metabolism. ORF27 encodes an adenylosuccinate synthetase that participates in purine biosynthesis by catalyzing the GTP-dependent conversion of inosine monophosphate to adenosine monophosphate [47]. Notably, this enzyme is located at the same loci of VP2 (VP2p26) and VP5 (VP5_gp26) chromosomes, and the presence of this enzyme constitutes one of the main distinguishing characteristics of the proposed VP2-like subfamily [29].

Recent studies have shown that some phages can employ alternative pathways of the classical holin-endolysin lysis system by employing the host cell secretion machinery to deliver their endolysins [48]. Our observations showed that ØVC8 is a virulent phage with lytic activity against several *V. cholerae* O1 strains; however, none of the identified genes of the ØVC8 genome appear to be involved in bacterial lysis. One possible explanation for the lytic activity of ØVC8 is that this phage uses a lysis pathway that differs from the classic system.

Comparative genome analysis of ØVC8, VP2, and VP5 showed similar genome sequences and genetic organization. The presence of an adenylosuccinate synthetase and the lack of a lysis cassette are unique traits of these three phages. However, the genome of ØVC8 shows five insertion/deletions that have not been identified in the VP2 and VP5 genomes; these insertion/deletions are located mainly in the unknown function region and in the replication module (Additional file 2: Figure S1). The effect of these insertion/deletions on the phenotypes of VP2 and VP5 remain unknown, largely because of a lack of data regarding the characteristics of these phages.

Mass spectrometric analysis of the structural proteins of phage ØVC8 showed that these proteins are distributed among the packing and structural modules (ORFs 17, 14, 8, and 4), indicating that ØVC8 requires these four structural proteins for prophage assembly and potentially for initial host recognition. Additionally, ORF8, which encodes a protein of 36.1 kDa, was identified as one of the most abundant structural proteins, suggesting that this is a protein with a high copy number that is presumably the major capsid protein.

## Conclusions

The obtained results allow us to propose that ØVC8, which was identified in a non-endemic cholera area, is a new specific lytic phage for toxigenic *V. cholerae* O1 strains. Some of its features suggest that this phage could be considered a member of the VP2-like phage subfamily. Additionally, the presence of an Ig domain could confer to this phage the ability to adhere to different mucus substrates (including the human intestine), a situation that may influence the epidemiology of cholera. Certain features of phage ØVC8 may be employed as alternative tools for monitoring environmental populations of *V. cholerae* strains and suggest it as a potential candidate for phage therapy.

## Additional files

Additional file 1: Table S1. Strains used for the phage host range test. These strains were not susceptible to infection by any of the phages used in the assay. (DOC 50 kb) Additional file 2: Figure S1. Whole-genome comparisons of ØVC8 against VP2 (up) and VP5 (down) using the Easyfig 2.1 program [49]. Predicted genes and transcription direction are represented as block arrows. ORFs are colored according to gene function, as indicated by the legend at the bottom. The degree of sequence similarity is indicated by color intensity, indicating the nucleotide identity levels (from 64 to 100 %). The comparisons were performed using BLASTn. Insertion/deletions and identity levels lower than 64 % are indicated by dotted squares. (DOC 22 kb)
