# Supplementary material for: Genetic characterization of ØVC8 lytic phage for Vibrio cholerae O1
Source: Virol J. 2016 Mar 22;13:47. doi: 10.1186/s12985-016-0490-x (PMC4802629; doi:10.1186/s12985-016-0490-x)
Supplement: Additional file 1: Table S1. — Strains used for the phage host range test. These strains were not susceptible to infection by any of the phages used in the assay. (DOC 50 kb) [file 12985_2016_490_MOESM1_ESM.doc]

Table S1. Strains used for the phage host range test. These strains were not susceptible to infection by any of the phages used in the assay.

| Strain | ID | Source/Reference |
| --- | --- | --- |
| **2740** | *V. cholerae* O1 | México ^1^ |
| **87145** | *V. cholerae* O1 | Levine *et al*., 1988^1^ |
| 87167 | *V. cholerae* O1 | México ^1^ |
| 88229 | *V. cholerae* O139 | CDC Atlanta ^1^ |
| **88678** | *V. cholerae* O139 | México ^1^ |
| 88758 | *V. metschnikovii* | México ^1^ |
| 88845 | *V. parahaemolyticus* | México ^1^ |
| 88846 | *V. parahaemolyticus* | México ^1^ |
| 88859 | *V. parahaemolyticus* | México ^1^ |
| 89812 | *V. parahaemolyticus* | México ^1^ |
| 96558 | *V. parahaemolyticus* | México ^1^ |
| 97259 | *V. parahaemolyticus* | México ^1^ |
| 97276 | *V. parahaemolyticus* | México ^1^ |
| AI-1852 | *V. cholerae* O139 | 2 |
| C-19385 | *V. cholerae* O1 | 2 |
| DK – 238 | *V. cholerae* O1 | 1 |
| DK – 60 | *V. cholerae* O1 | 1 |
| E1_1 | *V. alginolyticus* | This study |
| E1_5 | *A. veronii* | This study |
| E1_6 | *V. alginolyticus* | This study |
| E1_7 | *A. veronii* | This study |
| E1_8 | *A. veronii* | This study |
| E1_9 | *V. alginolyticus* | This study |
| E3_1 | *V. cholerae* No-O1 | This study |
| E3_10 | *V. cholerae* No-O1 | This study |
| E3_2 | *V. cholerae* No-O1 | This study |
| E3_3 | *V. cholerae* No-O1 | This study |
| E3_4 | *V. alginolyticus* | This study |
| E3_5 | *V. cholerae* No-O1 | This study |
| E3_8 | *V. cholerae* No-O1 | This study |
| HB101 | *E. coli* | México ^1^ |

Strains used for initial phage isolation are in bold.

1. Strains isolated from different sources were provided by the Laboratory of Bacteriology, Departamento de Salud Pública, Facultad de Medicina, Universidad Nacional Autónoma de México.

2. Provided by Dr. Shah M. Faruque of the Centre for Food and Water Borne Diseases at the International Centre for Diarrhoeal Disease Research, Bangladesh (ICDDR).
