# Supplementary material for: Genetic characterization of ØVC8 lytic phage for Vibrio cholerae O1
Source: Virol J. 2016 Mar 22;13:47. doi: 10.1186/s12985-016-0490-x (PMC4802629; doi:10.1186/s12985-016-0490-x)
Supplement: Additional file 2: Figure S1. — Whole-genome comparisons of ØVC8 against VP2 (up) and VP5 (down) using the Easyfig 2.1 program [49]. Predicted genes and transcription direction are represented as block arrows. ORFs are colored according to gene function, as indicated by the legend at the bottom. The degree of sequence similarity is indicated by color intensity, indicating the nucleotide identity levels (from 64 to 100 %). The comparisons were performed using BLASTn. Insertion/deletions and identity levels lower than 64 % are indicated by dotted squares. (DOC 22 kb) [file 12985_2016_490_MOESM2_ESM.doc]

**SUPPLEMENTARY FIGURES**

**Figure S1.** Whole-genome comparisons of ØVC8 against VP2 (up) and VP5 (down) using the Easyfig 2.1 program [51]. Predicted genes and transcription direction are represented as block arrows. ORFs are colored according to gene function, as indicated by the legend at the bottom. The degree of sequence similarity is indicated by color intensity, indicating the nucleotide identity levels (from 64% to 100%). The comparisons were performed using BLASTn. Insertion/deletions and identity levels lower than 64% are indicated by dotted squares.
